# Evaluation of diagnostic accuracy of dual-energy computed tomography in patients with chronic thromboembolic pulmonary hypertension compared to V/Q-SPECT and pulmonary angiogram

**DOI:** 10.3389/fmed.2023.1194272

**Published:** 2023-06-22

**Authors:** Armin Schüssler, Quirin Lug, Nils Kremer, Sebastian Harth, Steffen D. Kriechbaum, Manuel J. Richter, Stefan Guth, Christoph B. Wiedenroth, Khodr Tello, Dagmar Steiner, Werner Seeger, Gabriele Anja Krombach, Fritz Christian Roller

**Affiliations:** ^1^Department of Diagnostic and Interventional Radiology, Justus-Liebig-University Giessen, Giessen, Germany; ^2^Member of the German Center for Lung Research, Giessen, Germany; ^3^Department of Internal Medicine, Universities of Giessen and Marburg Lung Center (UGMLC), Institute for Lung Health (ILH), Cardio-Pulmonary Institute, Giessen, Germany; ^4^Department of Cardiology, Kerckhoff Heart and Thorax Centre, Bad Nauheim, Germany; ^5^Department of Thoracic Surgery, Kerckhoff Heart and Thorax Centre, Bad Nauheim, Germany; ^6^Department of Nuclear Medicine, Justus-Liebig-University Giessen, Giessen, Germany; ^7^DZHK (German Centre for Cardiovascular Research), Frankfurt am Main, Germany

**Keywords:** CTEPH: chronic thromboembolic pulmonary hypertension, dual-energy CT (DECT), V/Q SPECT, iodine mapping, pulmonary angiogram

## Abstract

**Purpose:**

The relevance of dual-energy computed tomography (DECT) for the detection of chronic thromboembolic pulmonary hypertension (CTEPH) still lies behind V/Q-SPECT in current clinical guidelines. Therefore, our study aimed to assess the diagnostic accuracy of DECT compared to V/Q-SPECT with invasive pulmonary angiogram (PA) serving as the reference standard.

**Methods:**

A total of 28 patients (mean age 62.1 years ± 10.6SD; 18 women) with clinically suspected CTEPH were retrospectively included. All patients received DECT with the calculation of iodine maps, V/Q-SPECT, and PA. Results of DECT and V/Q-SPECT were compared, and the percent of agreement, concordance (utilizing Cohen's kappa), and accuracy (kappa^2^) to PA were calculated. Furthermore, radiation doses were analyzed and compared.

**Results:**

In total, 18 patients were diagnosed with CTEPH (mean age 62.4 years ± 11.0SD; 10 women) and 10 patients had other diseases. Compared to PA, accuracy and concordance for DECT were superior to V/Q-SPECT in all patients (88.9% vs. 81.3%; k = 0.764 vs. k = 0.607) and in CTEPH patients (82.4% vs. 70.1%; k = 0.694 vs. k = 0.560). Furthermore, the mean radiation dose was significantly lower for DECT vs. V/Q-SPECT (*p* = 0.0081).

**Conclusion:**

In our patient cohort, DECT is at least equivalent to V/Q-SPECT in diagnosing CTEPH and has the added advantage of significantly lower radiation doses in combination with simultaneous assessment of lung and heart morphology. Hence, DECT should be the subject of ongoing research, and if our results are further confirmed, it should be implemented in future diagnostic PH algorithms at least on par with V/Q-SPECT.

## Introduction

Chronic thromboembolic pulmonary hypertension (CTEPH) is defined as precapillary pulmonary hypertension (PH) with persistent perfusion defects and/or endovascular lesions after at least 3 months of anticoagulative therapy (1). Symptoms of CTEPH are non-specific and differentiation to other cardiovascular diseases is difficult. As a result, patients with CTEPH are usually identified at a late disease stage (2). The persistence of thrombotic material after pulmonary embolism leads to fibrotic obstruction of pulmonary arteries, which is compounded by secondary inflammation, cell proliferation, and vascular remodeling (3–5). Due to the increase of both mean pulmonary artery pressure (mPAP) and pulmonary vascular resistance (PVR), the long-term impairment of the right heart function arises, which has a poor prognosis and high mortality (6, 7). Without treatment, CTEPH leads to death within a few years (8).

Pulmonary endarterectomy (PEA), balloon pulmonary angioplasty (BPA), and medical therapies are treatment options for CTEPH. The choice of treatment depends on the eligibility of patients for surgery and the type of pulmonary artery lesion (operable or non-operable CTEPH depending on central or peripheral occlusion pattern). If surgery is possible, therapy outcomes will be excellent (9) as CTEPH is the only etiology of PH which is potentially curable by PEA (9–12). For patients with inoperable CTEPH, BPA is an emerging and excellent therapy option, as several study groups previously showed (13–15).

Since in many cases, the diagnosis is made at a later stage and confirmation is often difficult, non-invasive imaging plays an increasingly important role in CTEPH. At present, ventilation/perfusion scintigraphy (V/Q-SPECT, if available) is recommended in the initial workup of pulmonary hypertension to exclude CTEPH (3, 16). Patients with perfusion defects with preserved ventilation (mismatch) are referred to PH centers for further diagnostic workup, including computed tomography pulmonary angiography (CTPA), invasive pulmonary angiogram (PA), and right heart catheterization (RHC). CTPA is widely recommended and used, but CTPA alone cannot rule out CTEPH as its predictive value is limited in peripheral CTEPH.

On the other hand, CTPA can be supported by dual-energy computed tomography (DECT) techniques, which allow visualization of iodine distribution after administration of iodine-containing contrast material as an equivalent of pulmonary perfusion. Through this means, DECT merges anatomical and physiological data in only one examination. In previous studies, the beneficial effects of DECT compared to standard CTPA (17–19) and V/Q-SPECT are shown (20). Other study groups presented high concordance rates for DECT compared to V/Q-SPECT (21). Furthermore, typical iodine distribution patterns on DECT might help in the identification and diagnosis of different vascular and lung diseases (22).

To further investigate the great potential of DECT, our study aimed to assess the diagnostic accuracy of DECT compared to V/Q-SPECT in patients with suspected CTEPH with PA serving as the “gold standard.”

## Materials and methods

This study was approved by the local ethics committee and was conducted for a period of 3 years. All patients with clinically suspected CTEPH between January 2018 and January 2020, who underwent DECT, V/Q-SPECT, PA, and RHC within a time span of 2 weeks for diagnostic workup were included in the current study. After a review of inclusion and exclusion criteria, a total of 28 patients (18 women) with a mean age of 62.1 years ± 10.6 standard deviation (SD) were included in this retrospective cohort study (ranging from 45 to 86 years). In total, 26 patients had a history of pulmonary embolism, two of the patients did not have prior pulmonary embolism and underwent diagnostic for further clarification in the case of unclear clinical findings and symptoms. Moreover, all patients underwent RHC and 26 of the patients were diagnosed with PH, which was defined as an elevation of the mPAP >20 mmHg at rest according to the ESC/ERS guideline (1). A total of 13 included study patients were already part of a previously performed study and evaluation (20).

### DECT

Patients were examined using a third-generation DECT unit (Somatom Force, Siemens Healthineers, Erlangen, Germany) with a standardized examination protocol. Technical parameters were as follows: maximum field-of-view (FoV) 355 mm, first X-ray tube 90 kV with a maximum reference value of 60 mAs, second X-ray tube 150 kV with a tin filter and a maximum reference value of 46 mAs, and the iterative reconstruction level set to the strength level three of five (ADMIRE, Siemens Healthineers, Erlangen, Germany). Contrast material was administered *via* a cubital vein (cannula size at least 18G) with a constant flow rate of 3.5 ml per second. Bolus triggering was used, and 60 ml of high-concentration contrast material (Ultravist 370^®^, Bayer Vital, Leverkusen, Germany) was injected followed by a 50 ml bolus of normal saline. The region-of-interest (ROI) for the bolus triggering was placed in the pulmonary trunk, and a threshold of 220 HU, followed by a scan delay of 10 s was used for initiating the scan. Scans were performed with patients in a supine position with their arms above the head from cranial to the caudal direction in deep inspiration breath-hold. In addition to standard lung and soft tissue reconstructions (covering the lung apex to the diaphragm), iodine maps were reconstructed in coronal, sagittal, and transverse orientation with a slice thickness and an increment of 4 mm. For postprocessing and reconstruction of iodine maps, a dedicated software (SyngoVia/Dual Energy CT, Siemens Healthineers, Erlangen, Germany) was used.

### V/Q-SPECT

V/Q-SPECT scans were acquired with the patients in a supine position with their arms extended in a cranial direction. Ventilation scintigraphy in which aerosol (Tc99m-Technegas) was inhaled three times with the closed nose *via* the mouth was initially performed. Patients were examined using a 360° technique with 60 projections and a matrix of 128 × 128 pixels. The maximum projection duration was set to 60 s or 50.000 counts in anterior projection. The energy window was set to 140 keV with a width of 15%. Afterward, perfusion scintigraphy was performed. The radiopharmacon (Tc99-MAA) was administered via a cubital vein. Technical settings for perfusion scintigraphy were identical to ventilation scintigraphy except for the matrix, which was set to 64 × 64 pixels. The amount of Tc99-MAA had to be at least four times greater than the amount of inhaled aerosol. For both, ventilation and perfusion scintigraphy, images in coronal, sagittal, and transverse orientation were reconstructed.

### Pulmonary angiogram

In brief, a 6 French (F) introducer sheath was placed into the femoral vein. For pulmonary digital subtraction angiography (DSA), the right and left pulmonary arteries were catheterized selectively using a 5 F pigtail catheter.

Angiograms were acquired at 7 frames per second. Posterior–anterior and lateral projections were obtained; posterior–anterior projections were slightly angulated to improve the visibility of the central pulmonary artery of each lung. High-concentration iodine contrast material (Ultravist 370^®^, Bayer-Vital, Leverkusen, Germany) was injected at a flow rate of 18–20 ml/s.

### Image analysis

CTPA and iodine maps were analyzed by an experienced radiologist (more than 12 years of experience in cardiothoracic imaging) blinded to clinical data, prior imaging studies, and the results of V/Q-SPECT and PA. Scans were considered suspicious for CTEPH if perfusion deficits were present at least in one segmental (with simultaneous detection of thromboembolic material in CTPA) or two subsegmental lung territories in the iodine map and/or if thromboembolic material was detected in the pulmonary arteries, accompanied by dilation of the pulmonary trunk (>30 mm). Moreover, CT scans were checked for direct and indirect signs of small airway disease and emphysematous lesions, which might mimic peripheral perfusion defects in the absence of thrombotic material in CTPA.

V/Q-SPECT scans were analyzed by two nuclear medicine physicians (with 30 and 15 years of experience) in consensus and blinded to clinical data, prior imaging studies, and results of DECT as well as PA. Scans were considered suspicious for CTEPH if at least one segmental or two subsegmental mismatch perfusion deficits were present.

PA series were analyzed by two experienced radiologists (more than 10 years of experience) in consensus blinded to clinical data, prior imaging studies, and the results of V/Q-SPECT as well as DECT. The observers reviewed the main pulmonary arteries, the lobar arteries, and the segmental and subsegmental arteries, as well as the parenchymal and venous perfusion in each lobe of the lung (superior, medial, and inferior lobe in the right and superior and inferior lobe in the left lung). Occlusion and perfusion deficits were analyzed and delineated for segmental and subsegmental arteries.

Due to cardiac motion artifacts and low spatial resolution on V/Q-SPECT scans, lung segments 7 and 8 were combined in the evaluation so that a total of 18 lung segments were analyzed for each patient and all analyzed imaging modalities.

The DSA images were analyzed at a dedicated workstation (Leonardo DSA/DR VA 30A, Siemens Healthineers, Erlangen, Germany).

The final CTEPH diagnosis was established based on all previous imaging findings by the multidisciplinary CTEPH conference, consisting of PH-experienced physicians, PEA surgeons, BPA interventionalists, and radiologists.

### Statistical analysis

Statistical analysis was performed using Prism GraphPad version 10 (GraphPad Software GmbH, San Diego, USA) and SPSS statistical software version 20 (SPSS, Chicago, Il, USA). Patient characteristics were described by mean ± SD. Data were tested for normal distribution using the Shapiro–Wilk test. For the normal distribution, Student's *t*-test was used, and for not normal distributed data, the Wilcoxon signed-rank test was used. In addition, the percent of agreement, concordance (*via* Cohen's kappa), and accuracy (*via* kappa^2^) between the different imaging modalities (DECT, V/Q-SPECT, and PA) were calculated for CTEPH and non-CTEPH patients on the lobe level and lung segment level. Cohen's kappa between 0 and 0.2 was interpreted as no agreement, between 0.21 to 0.39 as minimal agreement, between 0.40 and 0.59 as weak agreement, between 0.60 and 0.79 as moderate agreement, between 0.80 and 0.90 as strong agreement, and >0.90 as an almost perfect agreement. For comparison of radiation doses, the Wilcoxon signed-rank test was used. Equivalent doses were calculated by dose length product (DLP) for DECT, amount of administered radionuclide for V/Q-SPECT, and dose area products (DAP) for PA.

## Results

Of the 28 included patients (18 women; mean age 62.1 years ± 10.6 SD), 18 patients were diagnosed with CTEPH (10 women; mean age of 62.4 years ± 11.0 SD), and 10 patients were not diagnosed with CTEPH (8 women; mean age of 64.3 years ± 10.8 SD). Five patients without CTEPH were categorized as PH group 2 (PH due to left heart disease) according to the WHO classification, three patients as PH group 3 (PH due to emphysema), and one patient each with chronic thromboembolic disease (CTED) and with minor residuals after pulmonary embolism. Table 1 presents the patient demographics and an overview of the included diagnoses of the study cohort. All patients with CTEPH were correctly identified by DECT and V/Q-SPECT. Moreover, both the patient with CTED and the patient with minor residuals after pulmonary embolism were identified by DECT and V/Q-SPECT.

**Table 1:** Overview of diagnoses.

	***n***	**%**
**CTEPH**	18	64.3
**PH due to the left heart disease**	5	17.9
**Emphysema**	3	10.7
**CTED**	1	3.6
**Minor residuals after embolism**	1	3.6

CTEPH, chronic thromboembolic pulmonary hypertension; PH, pulmonary hypertension; CTPD without PH, chronic thromboembolic pulmonary disease without pulmonary hypertension.

A total of 140 lung lobes and 504 lung segments were examined regarding thromboembolism in 28 patients, including 90 lung lobes and 324 lung segments in 18 patients with CTEPH and 50 lung lobes and 180 lung segments in 10 patients without CTEPH.

PA, as the reference standard, depicted 202 segmental perfusion defects among the CTEPH patients and no perfusion defects in the non-CTEPH patients, representing 62.3 % of analyzed CTEPH segments and 40.1% of all analyzed segments. Compared with PA, DECT showed 175 lung segments with perfusion defects, of which 160 were classified as true positives and 15 as false negatives, whereas V/Q-SPECT revealed 185 segments with perfusion defects (145 true positives; 40 false positives). In the non-CTEPH patients, a complete concordance of DECT and V/Q-SPECT to PA was present.

In total, DECT showed a higher percentage of agreement, a higher concordance, and a better accuracy in contrast to V/Q-SPECT compared to PA for all patients (447 of 504 lung segments correct; accuracy 88.7%, sensitivity 79.2%, specificity 95.0%, positive predictive value 91.4% and negative predictive value 87.2%; k = 0.764 and kappa^2^ = 0.58 vs. 407 of 504 lung segments correct; accuracy 80.8%, sensitivity 71.8%, specificity 86.7%, positive predictive value 78.4%, negative predictive value 82.0%; k = 0.607 and kappa^2^ = 0.37). In patients diagnosed with CTEPH, the percentage of agreement, concordance, and accuracy for DECT against PA were also higher with 82.4%, k = 0.694, and kappa^2^ = 0.48 compared to V/Q-SPECT with 63.9%, k = 0.560, and kappa^2^ = 0.31.

In particular, DECT showed a strong concordance compared with PA for the right upper (k = 0.873; kappa^2^ = 0.762) and the right lower lobe (k = 0.874; kappa^2^ = 0.764), a moderate concordance for the left lower lobe (k = 0.784; kappa^2^ = 0.615), and a weak concordance for the left upper lobe (k = 0.564; kappa^2^ = 0.318) and the middle lobe (k = 0.563; kappa^2^ = 0.317). V/Q-SPECT showed moderate concordance for the right upper (k = 0.679; kappa^2^ = 0.461), right lower lobe (k = 0.694; kappa^2^ = 0.482), and the middle lobe (k = 0.604; kappa^2^ = 0.365) and a weak concordance for the left upper (k = 0.553; kappa^2^ = 0.306) and left lower lobe (k = 0.499; kappa^2^ = 0.249). Table 2 presents the percent of agreement, concordance, and accuracy for DECT and V/Q-SPECT compared to PA for all patients and CTEPH patients. Table 3 presents the sensitivities, specificities, and positive (PPV) and negative predictive values for DECT and V/Q-SPECT compared to PA for all patients.

**Table 2:** Concordance of DECT and V/Q-SPECT compared to PA.

	**DECT**			**V/Q-SPECT**		
	**% of agreement**	**Kappa (k)**	**Accuracy (kappa** ^2^ **)**	**% of agreement**	**Kappa (k)**	**Accuracy (kappa** ^2^ **)**
**All patients**	447/504 88.7%	0.764	0.58	407/504 80.8%	0.607	0.37
**CTEPH patients**	267/324 82.4 %	0.694	0.48	207/324 63.9%	0.56	0.31

PA, pulmonary angiogram; CTEPH, chronic thromboembolic pulmonary hypertension; DECT, dual-energy computed tomography; V/Q-SPECT, single photon emission computed tomography perfusion/ventilation; k, Cohen's kappa; Kappa^2^, accuracy.

**Table 3:** Sensitivity, specificity, negative predictive values, and positive predictive values of DECT, and V/Q-SPECT compared to PA.

	**Sensitivity**	**Specificity**	**PPV**	**NPV**
**DECT**	79.2 %	95.0 %	91.4 %	87.2 %
**V/Q-SPECT**	71.8 %	86.7 %	78.4 %	82.0 %

PA, pulmonary angiogram; CTEPH, chronic thromboembolic pulmonary hypertension; DECT, dual-energy computed tomography; V/Q-SPECT, single photon emission computed tomography perfusion/ventilation; NPV, negative predictive value; PPV, positive predictive value.

Table 4 presents the percentage of agreement, concordance, and accuracy of DECT and V/Q-SPECT compared to PA on the segmental level. The highest percentage of agreement, concordance, and accuracy on the segmental level between DECT and PA were achieved for segment 9 on the left side and segment 2 on the right side and between V/Q-SPECT and PA for segment 1 on the left side and segment 9 on the right side, whereas the lowest percentage of agreement, concordance, and accuracy was achieved for segment 2 on the left side and segment 4 on the right side for DECT and PA, and for segment 6 on the left side and segment 4 on the right side for V/Q-SPECT and PA.

**Table 4:** Percent of agreement, concordance, and accuracy of DECT and V/Q-SPECT to PA on the segmental level.

**Segment**	**DECT (%)**	**k**	**kappa^2^**	**V/Q- SPECT (%)**	**k**	**kappa^2^**
**L1**	89.27	0.746	0.556	89.29	0.779	0.607
**L2**	67.85	0.323	0.104	78.56	0.560	0.314
**L3**	89.29	0.761	0.579	75.00	0.443	0.196
**L4**	89.29	0.750	0.563	82.14	0.602	0.362
**L5**	89.29	0.750	0.563	89.29	0.761	0.579
**L6**	89.29	0.727	0.529	64.29	0.054	0.003
**L7/8**	89.29	0.781	0.610	71.43	0.407	0.166
**L9**	96.43	0.920	0.846	75.00	0.410	0.168
**L10**	92.86	0.689	0.475	78.57	0.533	0.284
**R1**	89.29	0.761	0.579	89.29	0.761	0.579
**R2**	100.00	1.000	1.000	82.14	0.632	0.399
**R3**	92.86	0.845	0.714	82.14	0.620	0.384
**R4**	75.00	0.484	0.234	75.00	0.495	0.245
**R5**	82.14	0.639	0.408	85.71	0.713	0.508
**R6**	92.86	0.836	0.699	82.14	0.602	0.362
**R7/8**	89.29	0.786	0.618	78.57	0.574	0.329
**R9**	92.86	0.858	0.736	92.86	0.858	0.736
**R10**	96.43	0.928	0.861	82.14	0.639	0.408

DECT, dual-energy computed tomography; V/Q-SPECT, single photon emission computed tomography perfusion/ventilation; k, Cohen's kappa; Kappa^2^, accuracy.

**Figure 1:**
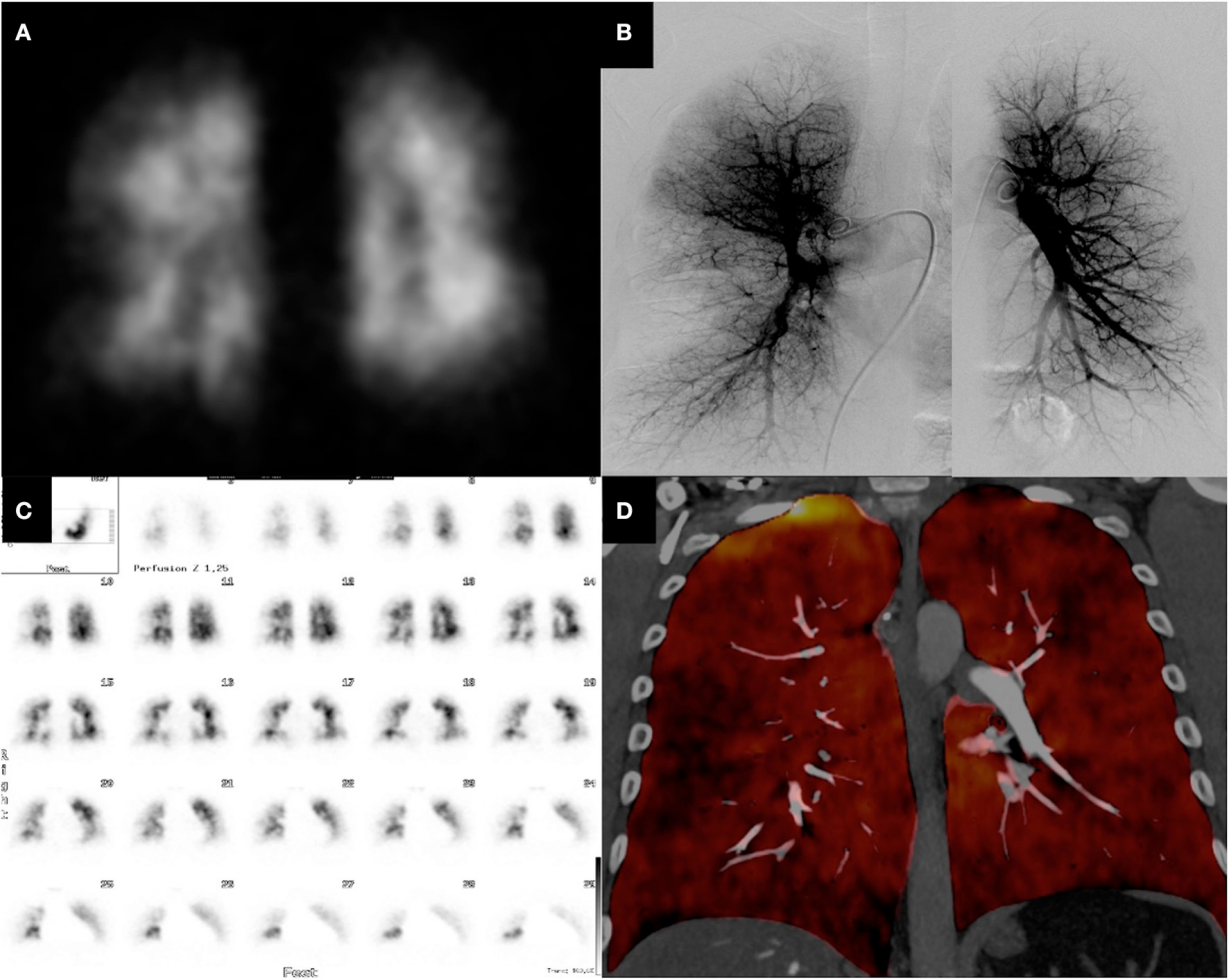
CTEPH patient with perfusion defects—DECT vs. V/Q-SPECT and PA. The figure shows the DECT **(D)**, V/Q-SPECT **(A, C)**, and PA **(B)** images of a 47-year-old male patient. All modalities show very clearly the segmental, subsegmental, and peripheral perfusion deficits.

**Figure 2:**
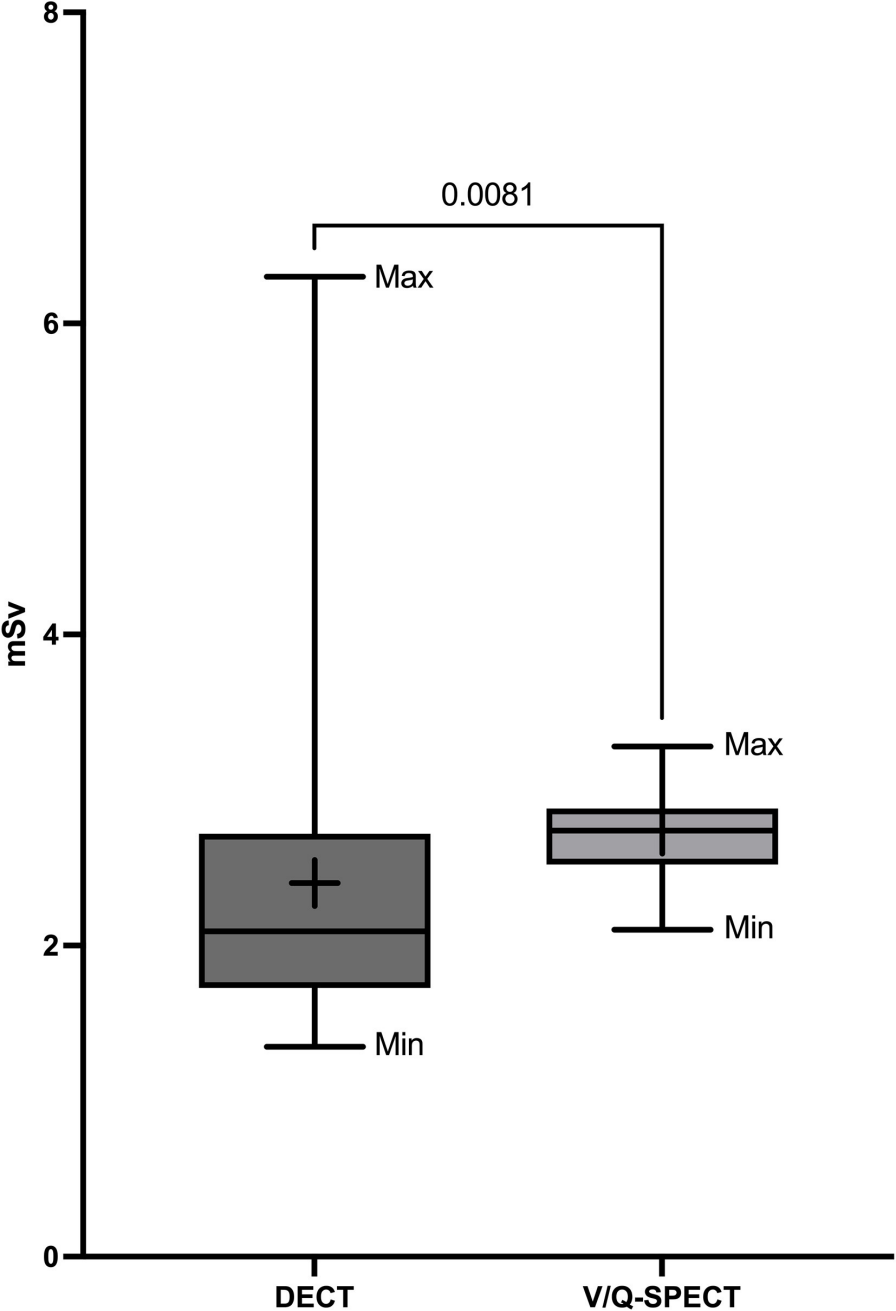
Radiation doses of DECT and V/Q-SPECT. The figure presents the mean radiation doses DECT and V/Q-SPECT as boxplots including minimum (Min), maximum (Max), box (from first to third quartile), median (horizontal line), the average value (+), and a *p*-value.

Figure 1 shows representative DECT, VQ-SPECT, and PA images of a CTEPH patient. The mean radiation dose for DECT was 2.40 mSv ± 1.03 SD and was significantly lower compared to V/Q-SPECT with a mean radiation dose of 2.74 mSv ± 0.26 (*p* = 0.0081) and PA with a mean radiation dose of 5.42 mSv ± 3.87 mSv (*p* < 0.0001). Figure 2 presents the radiation doses of DECT and V/Q-SPECT using boxplots.

## Discussion

V/Q-SPECT enables visualization of peripheral perfusion deficits, while CTPA is known to be limited to the presentation of thromboembolic material in central, segmental, and less also subsegmental pulmonary arteries. In contrast to CTPA, DECT allows visualization of perfusion in the lungs via reconstruction of iodine maps, and distal CTEPH is better visualized (23).

In recent years, some studies have already shown the promising potential of DECT in the diagnostic cascade of CTEPH. In particular, the beneficial effects of DECT compared to standard CTPA (17, 18), and even non-inferiority to V/Q-SPECT have already been demonstrated (20). To further validate the diagnostic capabilities of DECT, we compared DECT and V/Q-SPECT in patients with clinically suspected CTEPH against PA as the reference standard (“gold standard”) for the diagnosis of pulmonary arterial vasculature. However, to the best of our knowledge, this is the first study in this field to investigate this issue.

The two most significant findings of our study are as follows:

Compared to PA, DECT had a higher accuracy and agreement for the detection of segmental obstruction or perfusion defects over V/Q-SPECT in our patient cohort.

Moreover, radiation doses were significantly lower for DECT compared to V/Q-SPECT.

In line with the results of previously published studies, our results again highlight the high accuracy of DECT compared to V/Q-SPECT for diagnosing CTEPH (20). Our comparison of both techniques versus pulmonary angiography as the reference standard suggests that DECT is at least as good as V/Q-SPECT in diagnosing CTEPH. Nevertheless, it remains to be noted that both techniques are very reliable and detected correctly all CTEPH patients in our study despite differences in segmental perfusion assessment.

Compared to the recently published radiation dose values for DECT and V/Q-SPECT by the Fleischner Society in their position paper on imaging of pulmonary hypertension in adults (24), the radiation doses for both techniques were within the stated limits. Furthermore, compared to the S1 guideline of the German Society of nuclear medicine on lung scintigraphy (25), the radiation doses for DECT were significantly lower compared to V/Q-SPECT.

The significantly lower radiation dose of the DECT examinations in our patient cohort might be explained, on the one hand, by the use of the latest dual-source CT generation system with additional tin filtering and, on the other hand, by patient-specific factors such as the body mass index (BMI). It is well-known that radiation doses of CT examinations are more dependent on body constitution and body weight than V/Q-SPECT examinations.

However, in addition to its high diagnostic accuracy and low radiation dose, DECT offers many other important advantages as it simultaneously provides visualization of cardiac and vascular morphology—diameters of the pulmonary trunk and pulmonary arteries, ventricular and atrial dimensions, shunt vessels and malformations—and also lung morphology—emphysema, interstitial lung diseases, inflammation, and tumor. Another argument for the increased use of DECT is that DECT is significantly less time-consuming compared to V/Q-SPECT. The required time for V/Q-SPECT examinations lies between 25 and 30 min (25), whereas DECT examinations approximately take < 5 min.

All the described advantages and capabilities thus make DECT increasingly a kind of one-stop-shop examination in the clarification of pulmonary pathologies. In addition to the more commonly used DECT technique, subtraction CT can also be used for the reconstruction of iodine maps. In contrast to DECT, subtraction CT requires only motion correction software but no special hardware with two X-ray tubes, making subtraction CT somewhat simpler and less costly to implement clinically.

In terms of diagnostic performance, the two techniques do not differ similar to the results shown by Grob et al. (26).

This study has some limitations. First, our study was performed as a retrospective single-center cohort study with a small number of patients. Second, intra- and inter-observer agreements were not investigated for the different imaging modalities. However, this study offers a complex study design with standardization and implementation of V/Q-SPECT, DECT, and PA within only 14 days in a clinical setting at a dedicated German PH center, and interobserver agreement for DECT was previously reported as strong (24, 27). Moreover, high concordance rates of DECT and V/Q-SPECT have also been previously reported (21). Third, unfortunately, the study cohort did not include patients with pulmonary arterial hypertension (group 1), which represents the main differential diagnosis of patients with peripheral CTEPH (28). Otherwise, it has already been demonstrated that the detection of peripheral CTEPH was upgraded by perfusion imaging (29) and that the pattern of DECT perfusion changes can help to differentiate between both entities with high concordance to scintigraphy as previously demonstrated (28). Fourth, interpreting the different imaging modalities (DECT, V/Q-SPECT, and PA) is highly dependent on the investigator's experiences and expertise. However, in our study, all investigators had a high level of expertise and many years of experience at a qualified German PH center.

## Conclusion

Based on our results, we conclude that DECT, which can be performed with low radiation doses, is at least equivalent to V/Q-SPECT for diagnosing CTEPH. Therefore, DECT should be implemented in future diagnostic PH algorithms at least on par with V/Q-SPECT. Moreover, DECT possesses the potential to overtake V/Q-SPECT in diagnostic algorithms in dependence on the wider availability of new-generation CT systems as it enables simultaneous assessment of cardiac, vascular, and pulmonary morphology, which all make DECT a one-stop-shop modality. Further research in multicenter large-scale trials is warranted to verify our findings.

## Data availability statement

The raw data supporting the conclusions of this article will be made available by the authors, without undue reservation.

## Ethics statement

The studies involving human participants were reviewed and approved by Ethics Committee Department of Medicine Giessen/Justus-Liebig-University. Written informed consent for participation was not required for this study in accordance with the national legislation and the institutional requirements.

## Author contributions

FR, QL, AS, and GK were involved in study conception and design. FR, QL, AS, DS, SH, and GK contributed to acquisition of data. FR, QL, AS, NK, SH, SK, MR, SG, CW, KT, DS, WS, and GK helped in analysis and interpretation of data. FR, AS, NK, SH, SK, MR, SG, CW, KT, DS, WS, and GK helped in drafting of manuscript and involved in critical revision. All authors have read and approved the final manuscript.
